# A Robust Circular RNA-Associated Three-Gene Prognostic Signature for Patients with Gastric Cancer

**DOI:** 10.1155/2021/6633289

**Published:** 2021-04-21

**Authors:** Yang Li, Rui Li, Xiuli Wang, Yuan Yuan, Yangmei Zhang

**Affiliations:** ^1^Department of Central Laboratory, Xuzhou Central Hospital, Clinical School of Xuzhou Medical University, Xuzhou 221009, China; ^2^Department of Medical Oncology, Xuzhou Central Hospital, Clinical School of Xuzhou Medical University, Xuzhou 221009, China

## Abstract

Accumulating evidence has demonstrated that circular RNAs (circRNAs) play vital roles in cancer progression. However, the underlying molecular mechanisms of circRNAs remain poorly elucidated in gastric cancer (GC). The main purpose of present study is to explore the underlying regulatory mechanism by constructing a circRNA-associated competitive endogenous RNA (ceRNA) network and further establish a robust prognostic signature for patients with GC. Based on expression data of circRNA, microRNA, and mRNA derived from Gene Expression Omnibus (GEO) and The Cancer Genome Atlas (TCGA) databases, a circRNA-associated ceRNA network, containing 15 cirRNAs, 9 microRNAs, and 35 mRNAs, was constructed using the Starbase database. Functional enrichment analysis showed that the ceRNA network might be involved in many cancer-related pathways, such as regulation of transcription from RNA polymerase II promoter, mesodermal cell differentiation, and focal adhesion. A protein-protein interaction network was constructed based on genes within the circRNA-associated ceRNA network. We found that six of ten hub genes within the PPI network were significantly associated with overall survival (OS). Thus, using the LASSO method, we constructed a three-gene prognostic signature based on TCGA-GC cohort, which could classify GC patients into low-risk and high-risk groups with significant difference in OS (HR = 1.9, 95%CI = 1.14‐3.2, and log-rank *p* = 0.001). The prognostic performance of the three-gene signature was verified in GSE15459 (HR = 1.9, 95%CI = 1.27‐3.0, and log − rank *p* = 2.2*E* − 05) and GSE84437 (HR = 1.5, 95%CI = 1.17‐2.0, and log − rank *p* = 6.3*E* − 04). Multivariate Cox analysis further revealed that the three-gene prognostic signature could serve as an independent risk factor for OS. Taken together, our findings contribute to a better understanding of the underlying mechanisms of circRNAs in GC progression. Furthermore, a robust prognostic signature is meaningful to facilitate individualized treatment for patients with GC.

## 1. Introduction

Gastric cancer (GC) has been well known as one of the most malignant tumors with high incidence and mortality worldwide, which is responsible for over 1,000,000 new cases and 780,000 deaths predicted each year, making it ranks the fifth most frequently diagnosed cancer and the third leading cause of cancer-related death [1]. Despite recent improvements in comprehensive treatment of GC, the 5-year overall survival (OS) and disease-free survival (DFS) rate remain unsatisfactory, which has been largely attributed to the lack of efficient screening programs and a high frequency of recurrence and metastasis [2]. In clinical practice, the current American Joint Committee on Cancer (AJCC) TNM stage system has shown valuable but insufficient information for prognosis and estimation for GC patients [3]. Recently, several novel molecular classification schemas have been proposed according to the heterogeneous molecular characteristics [4–6]. Therefore, more efforts are needed to explore the molecular pathogenesis of GC and identify reliable prognostic molecular biomarkers, which can contribute to improving the understanding of GC progression and performing appropriate and individualized therapies.

In the last few years, along with an extensive characterization of the protein-coding genome in gastric cancer, increasing attention has been focused on circular RNAs (circRNAs), which are a class of endogenous noncoding RNAs characterized by covalently closed loop structures. circRNAs are abundant and stable in expression, and many of them are evolutionary conserved in many tissues [7, 8]. Accumulating evidence has demonstrated that the dysregulation of circRNAs could play critical roles in the initiation and progression of cancer [9, 10]. Hsiao et al. reported that circCCDC66 was upregulated in all stages of colon cancer and negatively correlated with prognosis, highlighting a novel oncogenic function in cancer progression and metastasis [11]. The research performed by Yao et al. demonstrated that circRNA_100876 expression was significantly elevated in non-small-cell lung cancer tissues and was closely associated with lymph node metastasis and tumor-node-metastasis stage, indicating that circRNA_100876 may be a potential cancer marker of patients with non-small-cell lung cancer [12]. Currently, there are few reports describing the role of circRNAs in GC. The biological function and regulatory mechanism of circRNAs in GC remain poorly elucidated and require further investigation.

Multiple properties of circRNAs have been identified in recent years, among which the role of “miRNA sponges” was most frequently discussed since some circRNAs possess miRNA response elements (MREs) [13, 14]. circRNAs sequester miRNAs to terminate the regulation of their target genes acting as competing endogenous RNA (ceRNA), promoting the cancer initiation, progression, and chemoresistance [15–17]. For example, Song et al. validated that upregulation of TPX2 by hsa_circRNA_101996-mediated inhibition of miR-8075 contributed to cervical cancer proliferation, migration, and invasion [18]. In addition, Yu et al. found that hsa_circ_0001445 could promote the expression of TIMP3, a well-known tumor suppressor, by sponging miR-17-3p and miR-181b-5p and further showed that hsa_circ_0001445 inhibits the growth and migration of hepatocellular carcinoma cells in vitro and in vivo data, providing a fresh perspective on circRNAs in hepatocellular carcinoma progression [19]. As comprehensive analysis of circRNAs remains insufficient for GC patients, the circRNA-miRNA-mRNA competing endogenous RNA network may provide an effective way to understand the regulatory mechanism and guide the individualized therapies.

In this study, by comprehensively integrating expression data of circRNAs, miRNAs, and mRNAs, the GC-related circRNA-miRNA-mRNA ceRNA network was established to explore the regulatory mechanism of key circRNAs potentially involved in GC progression. Moreover, we investigated the clinical relevance of genes within the ceRNA network and further developed a robust prognostic model for GC patients. This study provided a valuable insight for elucidating the regulatory mechanisms of circRNAs and constructing a reliable prognostic signature, which could guide individualized therapies and improve the clinical outcome for GC patients.

## 2. Materials and Methods

### 2.1. Data Processing

All cohorts and clinical information were described in Tables 1 and 2. circRNA expression profiles containing 5 pairs of GC and adjacent normal lung tissues were downloaded from the GEO (https://www.ncbi.nlm.nih.gov/geo/) database. The raw data were processed by background correction and quantile normalization. The expression data and clinical data of patients with GC were retrieved from TCGA (https://portal.gdc.cancer.gov/). Expression data included miRNA and mRNA expression levels for each patient, and clinical information included age, gender, pathological stage, lymph node metastasis, survival status, and overall survival time. The normalized count values of level 3 gene expression data derived from Illumina HiSeq V2 were extracted as gene expression measurement. Only patients with both survival information and expression data were included in this study. Ultimately, 345 patients were retained in our study. Two independent cohorts collected from GEO were used to test the prognostic ability. The GSE15459 and GSE84437 series contained 192 and 433 patients with both gene expression and clinical information, respectively. For expression data generated by the Affymetrix platforms, the Robust Multi-array Average algorithm was used for preprocessing the raw data. For expression data generated by the Illumina microarray platform, the originally processed data were used. All gene expression measurements were log2 transformed.

### 2.2. Construction of the circRNA-Associated ceRNA Network

Differentially expressed circRNAs (DEcircRNAs) were identified by the Student *t*-test with *p* < 0.05 and ∣logFC | >2 between GC and adjacent normal gastric tissues. As detected by long and short probes (the two kinds of probe were named CBC1 and CBC2, respectively), common DEcircRNAs were selected to construct the ceRNA network. Differentially expressed miRNAs (DEmiRNAs) and differentially expressed mRNAs (DEmRNAs) were identified by edgeR package with the threshold set at an FDR < 0.05 and ∣log2FC | >2. To better understand the effect of circRNAs on mRNAs mediated by combination with miRNAs in GC, a ceRNA network was constructed based on DEcircRNAs, DEmiRNAs, and DEmRNAs. Human sequences of DEcircRNAs and DEmiRNAs were downloaded from the circBase (http://www.circbase.org/) and miRBase (version 21; http://www.mirbase.org/) databases, respectively. The miRanda prediction tool was used to predict the interactions between DEcircRNAs and DEmiRNAs. Moreover, mRNAs targeted by the DEmiRNAs were retrieved from the Starbase (http://starbase.sysu.edu.cn/) database which provides the prediction results of seven predicted programs (TargetScan, microT, miRmap, picTar, RNA22, PITA, and miRanda). The interactions between miRNAs and mRNAs were selected if they were predicted in ≥3 programs. The target mRNAs were then overlapped with the DEmRNAs. Ultimately, removing the nodes that could not form a circRNA-miRNA-mRNA axis, a circRNA-associated ceRNA network was established and visualized by the Cytoscape software (version 3.7.0; http://www.cytoscape.org).

### 2.3. Functional Enrichment Analysis

In order to investigate the biological processes that the circRNA-associated ceRNA network may be involved in, we selected the DEmRNAs within the ceRNA network and further performed functional enrichment analysis using Database for Annotation, Visualization and Integrated Discovery (DAVID; http://www.david.abcc.ncifcrf.gov/). DAVID offers systematic and integrative functional annotation tools to unravel biological meaning behind a large list of genes. Gene Ontology (GO) contains three categories: biological processes, molecular function, and cellular components. Kyoto Encyclopedia of Genes and Genomes (KEGG) contains information about genomes, chemical substances, biological pathways, and diseases. *p* < 0.05 was regarded as statistically significant.

### 2.4. Establishment of the Protein-Protein Interaction (PPI) Network

PPI analysis is essential for illustrating the molecular mechanisms of key cellular activities in carcinogenesis. The Search Tool for the Retrieval of Interacting Genes/Proteins (STRING) database (https://string-db.org/) was used to evaluate the PPI information and further construct a PPI network based on DEmRNAs. An interaction score of 0.4 was regarded as the cutoff criterion, and the PPI network was visualized by the Cytoscape software. Furthermore, the univariate Cox regression analysis was used to evaluate the association with clinical outcome.

### 2.5. Construction of Prognostic Signature for Patients with GC

Based on DEmRNAs within the PPI network, we constructed a prognostic signature by the Least Absolute Shrinkage and Selection Operator (LASSO) method to achieve risk classification for GC patients. The LASSO regression is a popular method for variable selection in fitting high-dimension generalized linear model, which can get a more refined model by constructing a penalty function to reduce the variable numbers and effectively avoid overfitting. The glmnet package in R was utilized to perform the LASSO algorithm. Combining regression coefficient with corresponding gene expression values, a risk scoring model was established. The risk scores were calculated as shown in the following equation: Risk score = expression of gene 1∗*β*1 + expression of gene 2∗*β*2 + ⋯expression of gene i∗*β*i. *β*i is the regression coefficient of gene i, which represents the contribution of gene i to the prognostic risk score. After calculating the risk scores, patients in each cohort were classified into low-risk or high-risk group correspondingly using the median risk score as the cutoff point.

### 2.6. Statistical Analysis

The Kaplan-Meier method was used to assess the differences in survival time of low- and high-risk GC patients, and the log-rank test was used to determine the statistical significance of observed differences between groups. Multivariable Cox regression analysis and stratification analysis were used to assess whether the risk score was independent of other clinical features, such as stage, age, and gender. Hazard ratios (HRs) and 95% confidence intervals (CIs) were computed based on the Cox regression analysis. The difference in mortality rate between different risk groups was tested by Fisher's exact test. *p* < 0.05 was regarded as statistically significant.

## 3. Results

### 3.1. Identification of Differentially Expressed RNAs

**Figure 1:**
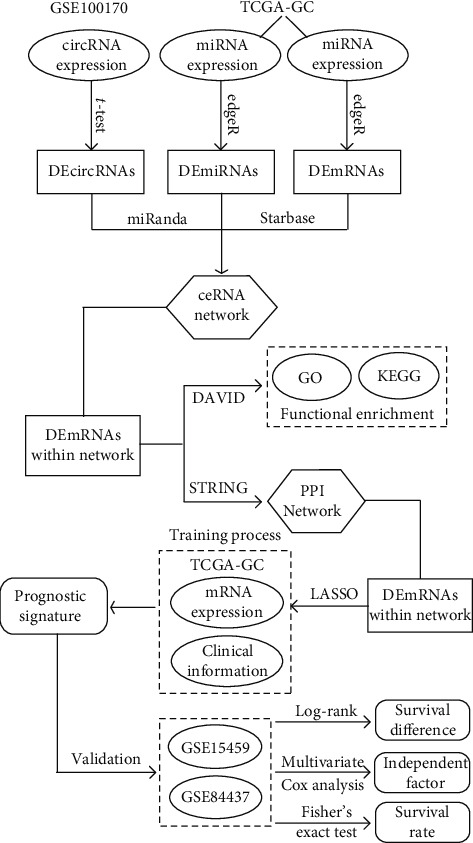
The flowchart for this study.

**Figure 2:**
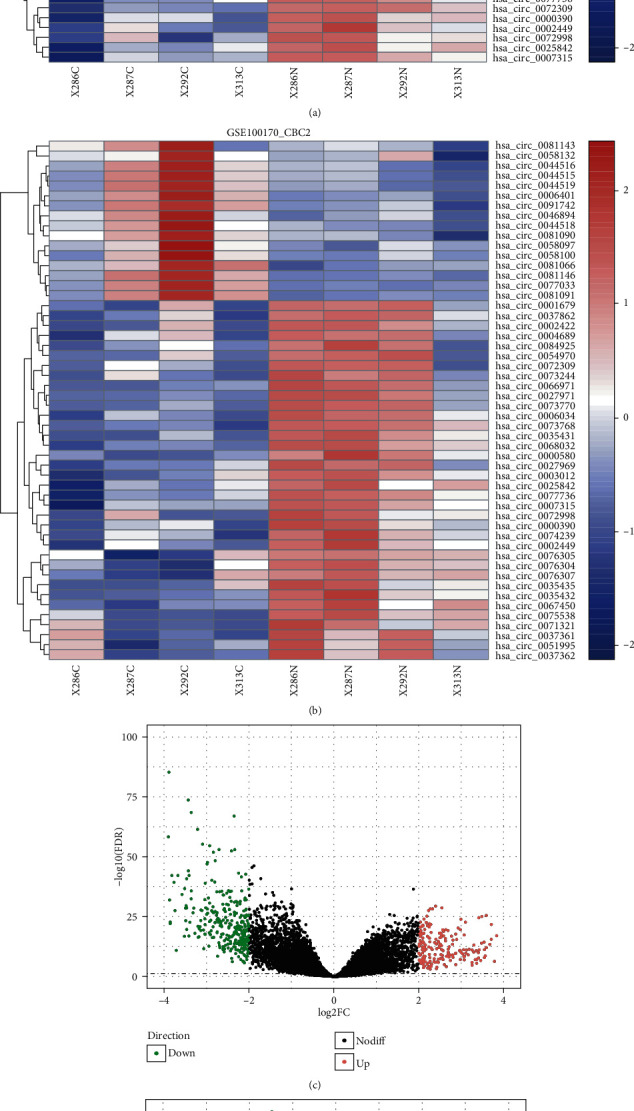
Heat maps of differentially expressed circular RNAs based on long ((a) CBC1) and short probes ((b) CBC2) and volcano plots of (c) differentially expressed mRNA and (d) differentially expressed miRNA. Red and green dots represent significantly upregulated and downregulated RNAs, respectively (FDR < 0.05 and ∣log2FC | >2.0).

The flowchart for this study is shown in Figure 1. Using a cutoff threshold of ∣log2FC | >2 and *p* < 0.05, a total of 52 circRNAs were differentially expressed between GC and non-GC tissues by both long and short probes (the two kinds of probe were named CBC1 and CBC2, respectively, Figures 2(a) and 2(b)), among which 16 were upregulated and 36 were downregulated. The concordance score was 100% (binomial test, *p* < 0.001). Using the “edgeR” package in R, we analyzed the expression data of miRNAs and mRNAs download from TCGA to select DEmiRNAs and DEmRNAs. With cutoff criteria of FDR < 0.05 and |log2FC | >2, 22 DEmiRNAs (including 11 upregulated and 11 downregulated miRNAs) and 520 DEmRNAs (including 210 upregulated and 310 downregulated mRNAs) were identified, respectively (Figures 2(c) and 2(d)).

### 3.2. circRNA-Associated ceRNA Network for GC

**Figure 3:**
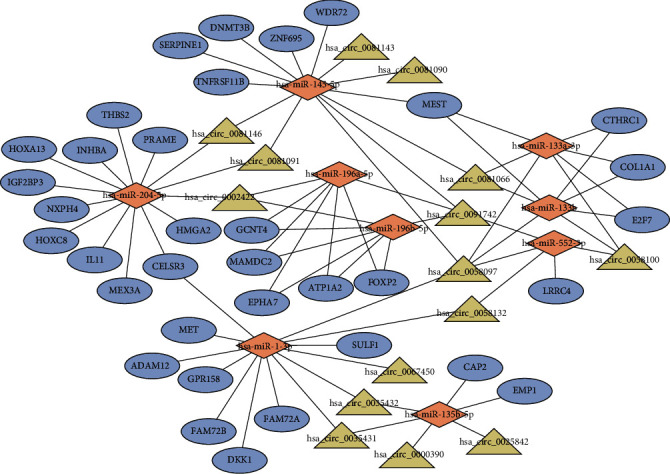
The circRNA-associated competing endogenous network in gastric cancer. Triangles represent circRNAs, diamonds represent miRNAs, ellipses represent mRNAs, and black lines represent circRNA-miRNA-mRNA interactions.

Using the miRanda prediction tool, 35 circRNA-miRNA interactions were predicted based on 17 DEcircRNA and 11 DEmiRNAs. We further identified DEmRNAs targeted by these DEmiRNAs from the Starbase database. A total of 46 miRNA-mRNA interactions were predicted, including 35 DEmRNAs predicted for 9 of the 11 DEmiRNAs. Integrating circRNA-miRNA interactions with miRNA-mRNA interactions, a circRNA-associated ceRNA network was established after removing the nodes that could not form a circRNA-miRNA-mRNA axis. This network contained 15 DEcirRNAs, 9 DEmiRNAs, and 35 DEmRNAs, as shown in Figure 3.

### 3.3. Functional Enrichment of DEmRNAs

**Figure 4:**
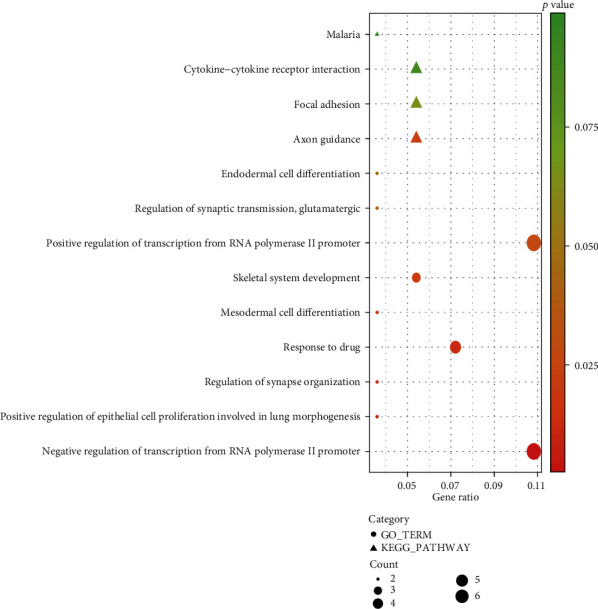
Enrichment of Gene Ontology (GO) terms and Kyoto Encyclopedia of Genes and Genomes (KEGG) pathways of differentially expressed mRNAs within the competing endogenous RNA network.

Using the DAVID database, DEmRNAs within the circRNA-associated ceRNA network were analyzed to explore the underlying functions and pathways which the ceRNA network might be involved in. The result is shown in Figure 4. GO annotation showed that DEmRNAs were significantly enriched in many cancer-related terms. For example, regulation of transcription from RNA polymerase II promoter, as the most significantly enriched term, has been reported to be closely related to the occurrence and progression of cancer. Another significantly enriched term, mesodermal cell differentiation, played key roles in endothelial cell development, which had been demonstrated to be associated with cancer progression and chemoresistance. From KEGG pathway enrichment analysis, axon guidance was significantly enriched by DEmRNAs. A couple of studies have suggested that dysregulation of genes within axon guidance pathway aid in the progression of pancreatic cancer and breast cancer. Besides, focal adhesion, as one of most common cancer-related pathways involved in tumor invasion and metastasis, was also enriched. Such results showed that DEmRNAs within the ceRNA network played crucial roles in multiple cancer-related processes, indicating that the circRNA-associated ceRNA network might be involved in GC invasion and progression.

### 3.4. Establishment of the PPI Network and Evaluation of the Prognostic Relevance

**Figure 5:**
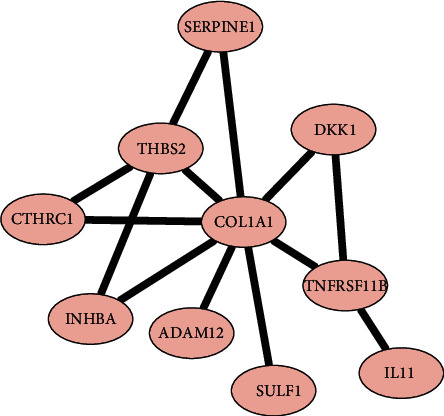
The protein-protein interaction network constructed by differentially expressed mRNAs within the competing endogenous RNA network.

Based on 36 DEmRNAs within the circRNA-associated ceRNA network, we explored the interactions among DEmRNAs using the STRING database. With cutoff criterion of interaction score > 0.4, 15 interactions were selected including 10 DEmRNAs after removing unconnected nodes. As shown in Figure 5, a PPI network was visualized by the Cytoscape software. Univariate cox regression analysis was performed to evaluate the association between gene expression and clinical outcome. The results showed that 6 of 10 DEmRNAs were significantly associated with OS, indicating the ability to prognosis for GC patients (Table 3).

### 3.5. Construction of the Prognostic Signature

**Figure 6:**
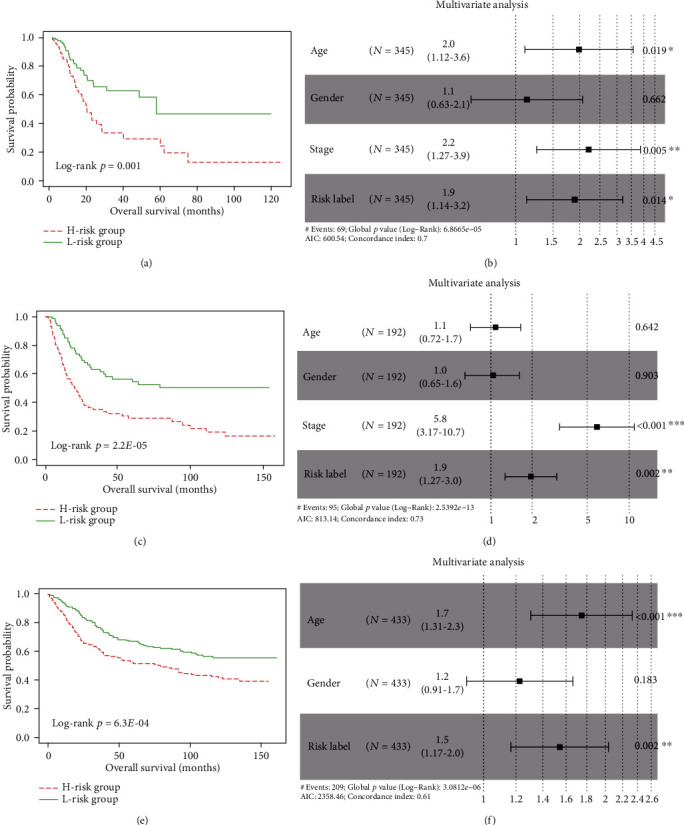
Prognostic performance of the three-gene prognostic signature. Kaplan-Meier curve of the overall survival between low-risk and high-risk groups in (a) TCGA-GC, (c) GSE15459, and (e) GSE84437, respectively; the forest maps calculated by multivariate Cox analysis in (b) TCGA-GC, (d) GSE15459, and (f) GSE84437, respectively.

Based on the prognostic ability of genes within the PPI network for GC patients, we tried to construct a prognostic signature based on the LASSO method using the R package “glmnet.” The degree of LASSO regression complexity is controlled by the parameter *λ* (0 < *λ* < 1). We obtained the optimal value of the parameter *λ* with the number of variables equal to three through multiple cross-validation. Therefore, combining the regression coefficients under the optimal *λ* value, we constructed a three-gene signature to guide the prognosis of GC patients. The risk-score formula was created as follows: Risk score = (0.088∗expression level of COL1A1) + (0.054∗expression level of DKK1) + (0.169∗expression level of SERPINE1). We calculated the risk score for each patient in TCGA-GC cohort. Patients were subsequently divided into a high-risk (*n* = 173) or a low-risk (*n* = 172) group according to the median risk score. K-M survival analysis showed that patients in high-risk group had significantly shorter OS than patients in low-risk group (log-rank *p* < 0.001; Figure 6(a)). After adjusting for clinical features including age, gender, and stage, the multivariate Cox regression analysis showed that the prognostic three-gene signature also had statistical significance as an independent prognostic factor in TCGA-GC training cohort (HR = 1.90, 95%CI = 1.14‐3.19, and *p* = 0.014; Figure 6(b)).

### 3.6. Validation of the Prognostic Signature

**Figure 7:**
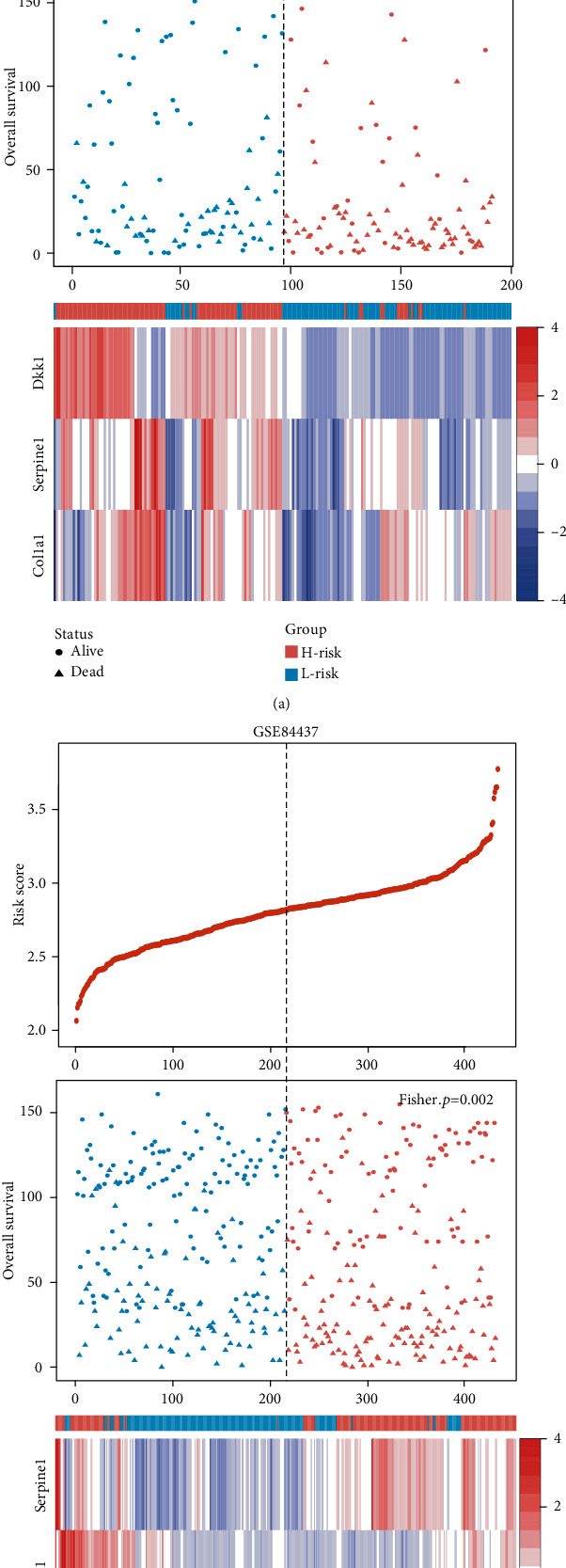
The risk score distribution, survival status, and expression profile of the three prognostic genes in (a) GSE15459 and (b) GSE84437 cohorts, respectively.

The prognostic performance of the three-gene signature was validated in two independent cohorts. In GSE15459 cohort, patients were divided into high-risk (*n* = 96) and low-risk (*n* = 96) groups with significant survival difference (log-rank *p* = 2.21*E* − 05; Figure 6(c)). Patients with high risk score had poorer OS. Besides, the mortality rate was 63.5% (61/92) in the high-risk group, significantly higher than 36.9% (34/92) in the low-risk group (*p* < 0.05, Fisher's exact test). Multivariate Cox regression analysis confirmed that the three-gene signature could serve as an independent prognostic factor for GC (HR = 1.95, 95%CI = 1.27‐2.99, and *p* = 0.002; Figure 6(d)). Similarly, each patient in GSE84437 cohort was classified into high-risk or low-risk group. We found that patients in high-risk group had a shorter survival time than patients in low-risk group (log-rank *p* < 6.32*E* − 04; Figure 6(e)). The mortality rate in high-risk group was significantly higher than that in low-risk group (55.8% vs. 40.7%, *p* < 0.05, Fisher's exact test). Besides, the multivariate Cox regression analysis also confirmed that the three-gene signature remained significantly associated with OS after adjusting for clinical features (HR = 1.54, 95%CI = 1.17‐2.03, and *p* = 0.002; Figure 6(f)). The risk score distribution, survival status, and expression profile of the three prognostic genes are shown in Figure 7.

## 4. Discussion

Gastric cancer is one of the leading causes of cancer-related mortality, and it has characteristically varying prognostic outcomes [20]. Emerging evidence shows that stable circRNAs play an increasingly important role in tumor progress, prognosis, and drug resistance [21–23]. In present study, based on expression data of circRNAs, miRNAs, and mRNAs, we screened aberrant RNAs and further constructed a circRNA-associated ceRNA network to investigate the regulatory mechanism for GC. To further evaluate the impact on clinical outcome, we performed the LASSO method to develop a robust three-gene prognostic model based on the circRNA-associated ceRNA network. Our results further highlight the important roles of circRNAs in GC and suggest potential therapeutic targets that warrant further investigation.

circRNAs are a new type of highly stable and abundant endogenous noncoding RNAs. With the development of high-throughput sequencing technique and bioinformatics analysis, circRNAs were found to function as ceRNAs to sponge miRNAs and then suppress their functions, indicating a novel mechanism for regulating miRNA activity and providing a promising mode of action for circRNAs. Recently, researchers continually focus on exploring the underlying biological mechanism for specific circRNAs involved in cancer occurrence and development [24, 25], especially gastric cancer [26]. In the current study, we constructed a circRNA-associated ceRNA network to make a systematic analysis for the regulatory mechanism of circRNAs related to GC progression. 15 circRNAs were characterized as core roles of GC progression in the circRNA-associated ceRNA network. For example, we found that hsa_circ_0081143 was significantly upregulated in GC tissues and predicted that hsa_circ_0081143 might regulate the expression of SERPINE1 as the sponge of miR-145 based on the circRNA-associated ceRNA network in our study. We further found that SERPINE1 was significantly related with OS, indicating the prognostic role of hsa_circ_0081143 for GC patients. Xue et al. confirmed that hsa_circ_0081143 was significantly upregulated in GC tissues, whose expression was closely association with advanced TNM stage, lymph node metastases, and poor overall survival of GC patients [27]. hsa_circ_0081143 silencing in vitro by siRNA can suppress GC cell viability and invasion ability and induce the sensitivity of GC cells to cisplatin (DDP) in vitro. Another circRNA, named hsa_circ_0058097, was also significantly upregulated in GC tissues. We found that hsa_circ_0058097 regulated multiple carcinogenic and prognosis-related genes, which might contribute to GC progression and prognosis by the regulatory axes, such as hsa_circ_0058097/hsa_miR_145-5p/SERPINE1, hsa_circ_0058097/hsa_miR_133a-3p/COL1A1, and hsa_circ_0058097/hsa_miR_1-3p/MET. Previous study had reported that hsa_circ_0058097 enhanced the expression of downstream target gene histone deacetylase 4 by sponge adsorption of miR-365a-5p, promoting tension-induced degeneration of endplate chondrocytes [28]. Besides, Fang et al. had reported that hsa_circ_0091742 was significantly upregulated in GC tissues [29]. In our study, we found that hsa_circ_0091742 was significantly upregulated in GC tissues. We predicted that hsa_circ_0091742 might bind to four miRNAs (including miR-145, miR-196a, miR-196b, and miR-552) and further regulate the expression of target genes related to GC progression and prognosis, such as SERPINE1, GCNT4, and EPHA7. Although other screened circRNAs have not been reported previously, the underlying regulatory mechanism was predicted by the circRNA-associated ceRNA network. More importantly, our research provided a guide for further experimental investigation to characterize the expression level and biological function of circRNAs in GC.

In present study, we further constructed a robust three-gene prognostic signature based on DEmRNAs involved in the circRNA-associated ceRNA network, which could be used as an independent indicator to make a risk classification for GC patients. We found that all three genes (SERPINE1, COL1A1, and DKK1) had been reported to be closely related to cancer progression. Lots of investigations have demonstrated the aberrant expression of SERPINE1 in various types of cancer [30, 31]. A recent article concluded that overexpression of SERPINE1 showed an activation effect on the phenotype of GC cells and EMT process, leading to a short overall survival for GC patients [32]. COL1A1, encoding the subunit of type I collagen, is the main constituent of the extracellular matrix (ECM) component in tumor microenvironment and plays critical role in cancer development and metastasis [33, 34]. Researchers found that COL1A1 expression was significantly upregulated in tumor tissues and was significantly associated with clinical outcome [35]. Moreover, knockdown of COL1A1 in gastric cancer cells curbed the proliferative, migratory, and invasive ability of cancer cells [36]. DKK1, a Wnt/ß-catenin pathway antagonist, has now emerged as an important regulator in a variety of human cancers. However, the role of DKK1 in cancer appears to be diverse. Many researches have suggested the tumorigenic effect of DKK1 [37, 38], while others have showed that DKK1 acts as a tumor suppressor [39, 40]. Lee et al. reported that overexpression of DKK1 in tissue and increased levels of DKK1 in serum were significantly associated with unfavorable prognosis in patients with GC [41]. In contrast, Jia et al. showed that the levels of DKK1 were decreased in serums and tissues of GC and restoration of DKK1 in tumor cells inhibited tumor cell growth and invasion [42]. In our study, we found that the DKK1 expression was significantly upregulated in tumor tissues and was significantly associated with overall survival. Furthermore, based on the circRNA-associated ceRNA network, all three genes might be regulated by multiple miRNAs and circRNAs. Results from our study showed that hsa_circ_0058097, as the hub node in the ceRNA network, might contribute to GC progression and prognosis by the regulatory axes, such as hsa_circ_0058097/hsa_miR_145-5p/SERPINE1, hsa_circ_0058097/hsa_miR_133a-3p/COL1A1, hsa_circ_0058097/hsa_miR_133b/COL1A1, and hsa_circ_0058097/hsa_miR_1-3p/DKK1. Further experimental investigation is needed to warrant these inferences.

## 5. Conclusions

To summarize, based on the expression data of circRNAs, miRNAs, and mRNAs, we developed a circRNA-associated ceRNA network to investigate the underlying regulatory mechanism in GC. Several important circRNAs were identified from the circRNA-associated ceRNA network, which might play crucial roles in GC progression. Moreover, we constructed a robust three-gene prognostic signature based on the DEmRNAs within the circRNA-associated ceRNA network, which could be used to make a risk classification for GC patients. This study provided a valuable insight for elucidating the regulatory mechanisms of circRNAs and constructed a reliable prognostic signature that could guide individualized therapies and improve clinical outcome for GC patients.

## Figures and Tables

**Table 1:** Cohorts analyzed in this study.

	GSE100170	TCGA-GC			GSE15459		GSE84437	
	circRNA	miRNA	mRNA	Clinic	mRNA	Clinic	mRNA	Clinic
Normal	5	41	32	-	-	-	-	-
Tumor	5	345	345	345	192	192	433	433
Platform	Agilent circRNA Array V1	Illumina HiSeq V2	Illumina HiSeq V2		Affymetrix U133 Plus 2		Illumina HT-12 V3	

**Table 2:** Clinical information analyzed in this study.

	TCGA-GC	GSE15459	GSE84437
Sample			
Normal	-	-	-
Tumor	345	192	433
Mean age (years; range)	65 (35-90)	64 (23-92)	60 (27-86)
Gender			
Male	219	125	296
Female	126	67	137
Stage			
I	49	31	-
II	110	29	-
III	149	72	-
IV	37	60	-
Status			
Alive	276	97	224
Dead	69	95	209
Platform	Illumina HiSeq V2	Affymetrix U133 Plus 2	Illumina HumanHT-12 V3

**Table 3:** Univariate Cox analysis of DEmRNAs within the PPI network.

DEmRNAs	HR	95% CI: low	95% CI: high	*p* value	DEdir
ADAM12	1.35	1.08	1.70	0.008	1
COL1A1	1.25	1.06	1.47	0.006	1
CTHRC1	1.23	1.04	1.45	0.016	1
DKK1	1.10	0.98	1.23	0.094	1
IL11	1.17	0.98	1.39	0.083	1
INHBA	1.26	1.03	1.54	0.027	1
SERPINE1	1.33	1.13	1.56	<0.001	1
SULF1	1.15	0.99	1.34	0.067	1
THBS2	1.16	1.01	1.34	0.044	1
TNFRSF11B	1.01	0.87	1.16	0.977	1

Note: DEmRNAs: differentially expressed mRNAs; HR: hazard ratio; CI: confidence interval; DEdir: differentially expressed direction.

## Data Availability

All data sets analyzed in this study were downloaded from the GEO (https://www.ncbi.nlm.nih.gov/geo/) and TCGA (https://portal.gdc.cancer.gov/) public databases.
